# Shared Decision‐Making on Late‐Term Gestation Management (LATE‐Study): A Cross‐Sectional Survey Among Maternity Care Providers

**DOI:** 10.1111/birt.70099

**Published:** 2026-08-02

**Authors:** Anna E. Seijmonsbergen‐Schermers, Kika Grosheide, Fatima Hammiche, Hannah de Klerk, Judit K. J. Keulen, Caroline J. Bax, Lilian L. Peters, Corine J. Verhoeven

**Affiliations:** ^1^ Amsterdam UMC Location Vrije Universiteit Amsterdam Midwifery Science Amsterdam the Netherlands; ^2^ Midwifery Academy Amsterdam Groningen, Inholland Amsterdam the Netherlands; ^3^ Amsterdam Public Health Quality of Care Amsterdam the Netherlands; ^4^ Department of Primary and Long‐Term Care University of Groningen, University Medical Center Groningen Groningen AD the Netherlands; ^5^ Department of Obstetrics and Gynecology Amsterdam UMC Location Amsterdam University Medical Center Amsterdam the Netherlands; ^6^ Amsterdam Reproduction and Development Amsterdam the Netherlands; ^7^ Zuyd University of Applied Sciences Research Center for Midwifery Science Maastricht the Netherlands; ^8^ Department of Obstetrics and Gynecology Maxima Medical Center Veldhoven the Netherlands

**Keywords:** guidelines as topic [MeSH], labor induced [MeSH], late‐term gestation, shared decision‐making [MeSH]

## Abstract

**Objective:**

Since 2021, a Dutch guideline recommends offering induction of labor (IOL) from 41 + 0 weeks, alongside the option of expectant management (EM), through shared decision‐making (SDM). We examined care providers' opinions and use of the guidelines' SDM tools, self‐reported adherence to SDM content recommendations, perspectives on SDM conversations, and its association with birth beliefs.

**Methods:**

For this cross‐sectional survey, including 575 maternity care providers, an online questionnaire was developed. Most questions used a 5‐point Likert scale. Birth beliefs (medical vs. natural) were assessed using the Birth Beliefs Scale. Uni‐ and multivariable regression analyses were used to assess the association between a medical and natural view and SDM content and perspectives.

**Results:**

Among participants aware of the tools, usage rates were 63% for Tool 1 (steps of SDM), 72% for Tool 2 (choice card IOL/EM), and 51% for Tool 3 (choice card alternatives). Helpfulness scores exceeded the neutral midpoint (3.7, 3.8, and 3.7). The majority of participants reported addressing most of the items of SDM that were recommended in the guideline, though lower rates were seen for addressing outcome percentages (37%), parity differences (33%), and EM beyond 42 weeks (37%). Birth beliefs influenced SDM content, emphasis on advantages of options, and perspectives; those with stronger natural views paid more attention to women's preferences and alternatives.

**Conclusion:**

Care providers are generally positive about the tools in the late‐term management guideline, though not all use them. Key elements for SDM are often omitted, and birth beliefs influence how SDM is conducted.

## 1 Introduction

In maternity care, choices increasingly need to be made, requiring women's involvement through shared decision‐making (SDM). SDM is a collaborative process involving both the care provider and the woman, aiming to base decisions on the best available evidence as well as the woman's preferences, values, and goals [1]. Stiggelbout et al. [2] outlined four steps in the SDM process, as illustrated in Textbox 1 [2].

Textbox 1 **The four steps of SDM of Stiggelbout et al. [2].**

(1) the professional informs the client that a decision is to be made and that the client's opinion is important.

(2) the professional explains the options and their pros and cons.

(3) the professional and the client discuss the client's preferences and the professional supports the client in deliberation.

(4) the professional and the client discuss the client's wish to make the decision, make or postpone the decision, and discuss follow‐up.

One area in which SDM is increasingly being utilized is induction of labor (IOL). However, literature on the content of SDM conversations regarding IOL is limited. The experiences of expectant parents with SDM during pregnancy and childbirth vary. They highly value being involved in decision‐making, which improves satisfaction, reduces distress, and fosters empowerment [3, 4]. SDM in relation to IOL is particularly relevant because the indication for IOL has recently been set at earlier gestational ages [5], and there is an ongoing debate about IOL at late‐term gestations [6, 7, 8, 9, 10, 11].

Since 2021, a new guideline on the management of late‐term gestations in the Netherlands has recommended offering pregnant women the option of IOL from 41 + 0 weeks, in addition to the option of expectant management (EM) up to 42 + 0 weeks [11]. The guideline also specifies the information that should be provided during the SDM conversation and recommends using choice cards to explain the impact of both options on perinatal outcomes. The guideline provided three tools:

1. Tool 1: BRAINS‐steps (see Textbox 1 and Supporting Information 2);
2. Tool 2: Choice card for IOL vs. EM (see Supporting Information 3);
3. Tool 3: Choice card with alternative options (see Supporting Information 4).

The first tool is a comprehensive tool for care providers that follows the steps of BRAIN—an acronym for Benefits, Risks, Alternatives, Intuition, Nothing [12]—with the addition of a new step, ‘Standstill’. The five steps of BRAINS for SDM are illustrated in Textbox 2.

Care providers' beliefs and opinions may sometimes differ from guideline recommendations. The publication of new guidelines does not automatically translate into implementation in daily practice [13, 14]. It is unknown to what extent care providers adhere to specific recommendations from national guidelines and utilize the tools provided, and how care providers' beliefs influence this adherence and their opinion on SDM.

Textbox 2 **The five steps of the BRAINS decision‐making tool in the national guideline concerning management of late‐term gestations for women with low‐risk pregnancies [11]:**

(B) Benefits:

– Percentages of outcomes for each decision option.
– Advantages of IOL.
– Advantages of EM.

(R) Risks:

– Disadvantages of IOL.
– Disadvantages of EM.
– Parity differences.

(A) Alternatives:

– Sweeping of the membranes.
– Artificially rupturing the membranes prior to the onset of labor.
– EM beyond 42 weeks.

(I) Intuition:

– Asking whether counseling is desired.
– Recognizing preferences of the pregnant woman (and her partner).
– Checking understanding.

(N) Nothing:

– Acknowledging not yet deciding as an option.

(S) Standstill:

– Giving time to think.
– Inviting questions.

Therefore, we first aimed to examine care providers' opinions on specific SDM tools in the national guideline on late‐term gestation management and how they are being used. Secondly, we sought to gain insight into the content of SDM conversations and the extent to which the SDM recommendations in the guideline are adhered to, and their association with care providers' beliefs. Thirdly, we aimed to explore the perspectives of care providers regarding these conversations in relation to late‐term management and their association with care providers' beliefs.

## 2 Methods

This study employed a cross‐sectional design and is part of the LATE‐term gestation management study (LATE‐study). The LATE study aims to comprehensively evaluate late‐term gestation management in the Netherlands (including women's experiences and variations in clinical protocols). This article, together with a previous publication [15], describes findings from one component of the study: An evaluation of care providers' perspectives. It targeted maternity care providers in the Netherlands. Inclusion criteria comprised primary care midwives, hospital‐based midwives, obstetricians, and medical residents. These care providers worked in either midwife‐led or obstetrician‐led settings. Women receiving midwife‐led care are generally at low risk of complications and are referred to obstetrician‐led care if complications or higher risks occur [16]. Primary care midwives work autonomously in the community, while hospital‐based midwives work within obstetrician‐led care. In general, IOL is conducted within obstetrician‐led care, with some exceptions. Exclusion criteria for this study included not having worked in maternity care for more than one year, not answering questions about SDM conversations, and completing less than 25% of the online questionnaire. The most important questionnaire items were placed at the beginning of the survey to ensure that responses reaching at least the 25% threshold captured the key variables of interest. Given the length of the questionnaire, duplicate completions beyond this threshold were considered unlikely.

### 2.1 Questionnaire

The development and distribution of the questionnaire has been described in a previous publication [15]. The online questionnaire was developed in collaboration between the authors of this study, care providers, and a client representative. Four semi‐structured interviews were held with midwives and obstetricians and analyzed using inductive thematic analysis to identify relevant themes for inclusion. Subsequent versions were reviewed by a working group, including care providers and a client representative, and piloted among twelve care providers and researchers, leading to the addition, removal, and clarification of several items. The questionnaire was subsequently disseminated to maternity care providers through professional organizations, newsletters, social media, and congresses, and is included in Supporting Information 1.

As the survey was openly accessible, no response rate could be calculated. The questionnaire aimed to assess care providers' perspectives and adherence to the guideline recommendations, to evaluate the content of SDM conversations, and to determine the utilization of the tools provided in the guideline. Informed consent was obtained at the start of the questionnaire, after giving general information about the study and the guideline that was the subject of this study.

### 2.2 Variables

Participants' characteristics were: Profession, gender, age, work experience, employment, knowledge about the scientific foundation of the guideline, frequency of having an SDM conversation on late‐term pregnancies, positive attitude towards guidelines in general, experience of a capacity problem, awareness of the guideline's existence, and degree to which the guideline was read. Some additional characteristics specifically apply to a profession. A characteristic specifically applying to obstetricians was: Their main speciality. A characteristic applying to obstetricians and obstetric residents was: Frequency of working at an obstetric unit. Characteristics applying to obstetrician‐led care were: Hospital type and hospital number of births per year. A characteristic applying to midwives was: Their highest level of education. Characteristics applying to primary care midwives were: Practice size and possibility of transfer to the hospital of first choice.

Last, birth beliefs were assessed as a characteristic, using the Birth Beliefs Scale (BBS). The Birth Beliefs Scale assessed care providers' views on childbirth, providing two scores: A medical and a natural score. The medical score reflects a belief about birth as a medical and risky process, and the natural score reflects a belief about birth as a natural and safe process. Ratings were based on a 5‐point Likert scale, where a higher score indicates stronger agreement with the corresponding view. The Dutch translation of this scale, which has been validated for use among care providers, includes eleven statements—five relating to the medical view and six to the natural view [17]. The scale is provided in Supporting Information 1.

There were three groups of outcome measures:

1. Care providers' awareness and use of the three tools included in the guideline and use of other tools (dichotomously).
2. Inclusion of the guideline‐recommended topics and items of SDM in care providers' SDM conversations, using the BRAINS framework, as recommended in the guideline (dichotomously). In the questionnaire, care providers were asked to indicate for each guideline‐recommended topic and item whether they discussed it in at least 75% of their SDM conversations.
3. Perspectives of care providers on statements regarding SDM (on a 5‐point Likert Scale).

### 2.3 Analyses

Characteristics of participants were presented as numbers and percentages for categorical variables, and as means with standard deviations for continuous variables. Characteristics specific to particular professions or groups were described only within those groups, while characteristics applicable to all participants were presented stratified by three professional groups (obstetrician/resident, hospital‐based midwife, and primary care midwife).

Several visualizations were used to describe the outcomes. Stratification was necessary because the three professional groups differ in their clinical context, making combined results difficult to interpret. Additionally, pooling the groups would skew the results towards the largest professional group in the survey. However, as we did not aim to compare professional groups, no statistical significance tests were performed for differences between groups.

To explore the associations between the medical and natural view scores of the BBS and the self‐reported topics/items discussed in SDM conversations, descriptive analyses were conducted to calculate the mean and standard deviation of both view scores for each topic/item. Subsequently, univariable and multivariable logistic regression analyses were used to estimate unadjusted and adjusted odds ratios of the association between the BBS and the topics/items in SDM conversations. In addition, to assess the relationship between the BBS and perspectives on SDM, univariable and multivariable linear regression analyses were performed, resulting in unadjusted and adjusted regression coefficients (β).

All data analyses were conducted using STATA version 14.1 (StataCorp, Texas, USA).

## 3 Results

### 3.1 Participant Characteristics

The questionnaire received 2531 entries. This was a large number because of the open access nature of the questionnaire. The entire questionnaire was completed by 494 care providers who conducted SDM conversations. A further 81 care providers completed at least 25% of the questionnaire, resulting in a total inclusion of 575 participants. This represented 11.6% of all working obstetricians and 11.2% of all working midwives in the Netherlands. Of the included participants, 134 (23%) were obstetricians, 40 (7%) residents, 108 (19%) hospital‐based midwives, and 293 (51%) primary care midwives (Table 1). Of the obstetricians, 77% were specialized in perinatology, and 90% worked in an obstetric unit on a weekly basis. Most care providers in obstetrician‐led care worked in secondary/peripheral centers (77%) and in hospitals with more than 2000 births annually (55%). Primary care midwives were most likely to work in practices with three to five midwives (58%).

**TABLE 1:** Participant characteristics (*n* = 575).

Variables	*n* (%) or mean [SD]
Gender	
Female	463 (92)
Male	34 (7)
Prefer not to answer	8 (1)
Age in years, mean [SD]	41 [11]
Work experience in years, mean [SD]	16 [10]
Employment	
Paid employment	194 (38)
Self‐employed without partnership	99 (20)
Partnership	202 (40)
Other	9 (2)
Knowledge about scientific foundation of the guideline, mean [SD]	3.8 [0.9]
Unsure (*n* (%))	12 (2)
Frequency of having a shared decision‐making conversation on late‐term pregnancies	
Never	15 (3)
Monthly or less	89 (15)
Bi‐weekly	174 (30)
Weekly	297 (52)
Positive attitude towards guidelines in general, mean [SD]	3.7 [0.8]
Unsure (*n* (%))	10 (2)
Experience of capacity problem*, mean [SD]	3.6 [0.8]
Unsure (*n* (%))	1 (0)
Birth Beliefs Scale	
Medical view, mean [SD]	2.8 [0.6]
Natural view, mean [SD]	3.9 [0.6]
Aware of existence of guideline	533 (93)
If aware of existence:	
Read further than recommendations	353 (66)
Did not read guideline	25 (5)
Profession	
Obstetrician	134 (23)
Obstetric resident	40 (7)
Hospital‐based midwife	108 (19)
Primary care midwife	293 (51)
Profession‐related variables	
Applying to obstetricians:	Total *n* = 123
Main specialty	
Perinatology	95 (77)
Urogynecology	5 (4)
Reproductive medicine	4 (3)
Oncology	2 (2)
Benign gynecology	6 (5)
General obstetrician	11 (9)
Applying to obstetricians and obstetric residents:	Total *n* = 155
Frequency working at an obstetric unit	
Never	1 (1)
Monthly	2 (1)
Bi‐weekly	13 (8)
Weekly	139 (90)
Applying to obstetrician‐led care:	Total *n* = 244
Hospital type	
Tertiary/academic center	57 (23)
Secondary/peripheral center	187 (77)
Hospitals’ number of births per year	
< 500	1 (0)
500–1000	18 (7)
1000–1500	38 (16)
1500–2000	46 (19)
> 2000	133 (55)
Unsure or missing	7 (3)
Applying to midwives:	Total *n* = 349
Highest level of education	
Bachelor degree	283 (81)
Master degree	56 (16)
PhD	10 (3)
Applying to primary care midwives:	Total *n* = 259
Practice size	
1–2 midwives	30 (12)
3–5 midwives	151 (58)
6–10 midwives	70 (27)
11 or more midwives	8 (3)
Possibility of transfer to hospital of first choice (on scale from 1 to 10), mean [SD]	6.8 [2.1]

*Note:* Number of missing: *n* = 70 for gender, *n* = 71 for age, working experience, country of education, and employment, *n* = 80 for region of work, *n* = 75 for knowledge about scientific foundation of the guideline, and positive attitude towards guidelines in general, *n* = 73 for experience of capacity problem, *n* = 79 for Birth Beliefs Scale, *n* = 0 for frequency shared decision‐making, aware of existence of guideline, read further than recommendations, did not read guideline, and profession (these questions were asked at the beginning of the survey, while the others were asked at the end); *n* = 11 for questions applying to obstetricians, *n* = 19 for questions applying to obstetricians and obstetric residents, *n* = 38 for questions applying to secondary care, *n* = 52 for questions applying to midwives, *n* = 34 for questions applying to primary care midwives.

* Measured on a 5‐point Likert scale.

The majority of participants were female (92%), with a mean age of 41 years and a mean of 16 years of work experience (Table 1). The majority of participants (52%) conducted SDM conversations weekly, with a further 30% doing so bi‐weekly. The general positive attitude towards guidelines was rated 3.7 on a 5‐point Likert scale, with obstetricians (4.1) and hospital‐based midwives (4.0) scoring higher than primary care midwives (3.4). Participants' awareness of the existence of the guideline was high (93%), though lower among hospital‐based midwives (86%). However, only 66% of the care providers who were aware of the guideline read beyond the recommendations, with obstetricians being more likely to report that they had read further (74%) than hospital‐based midwives (67%) and primary care midwives (61%).

### 3.2 Usage and Opinions of Tools

Awareness of the tools provided with the guideline (Figure 1) was lower than the awareness of the guideline itself, with 73% aware of Tool 1 (BRAINS), 81% aware of Tool 2 (choice card for IOL/EM), and 60% aware of Tool 3 (choice card alternatives). Only those who were aware of the existence of the tools were asked about their usage: 63% of participants used Tool 1, 72% used Tool 2, and 51% used Tool 3. Other tools, which were not included in the guideline, were used by 29% of participants, predominantly primary care midwives. Primary care midwives were more likely to use Tool 1 (72%) than hospital‐based midwives (56%) and obstetricians (52%), but less likely to use Tool 2 (66%) compared to hospital‐based midwives (71%) and obstetricians (81%). There were no large differences in opinions about the tools between the professional groups (Figure S5), although minor differences were observed that correlated with the usage patterns shown in Figure 1. The overall usefulness/helpfulness scores for the tools were above average, with Tool 1 scoring 3.7, Tool 2 scoring 3.8, and Tool 3 scoring 3.7.

**FIGURE 1:**
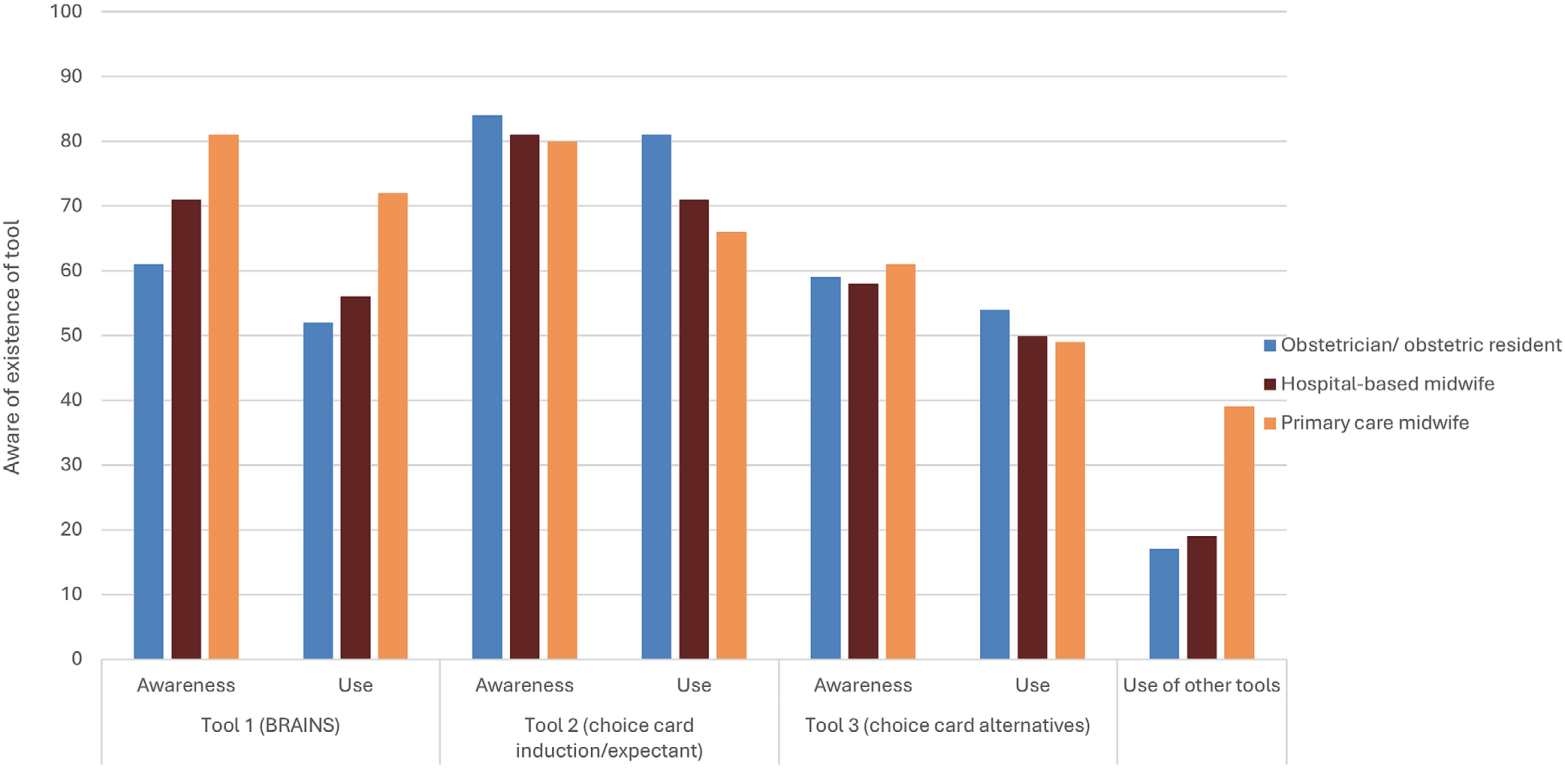
Care providers' awareness and use of the tools* provided with the guideline (dichotomously), stratified by profession (*n* = 574). Legend: *Tools in the guideline: Tool 1: Tool for care providers including BRAINS‐elements. Tool 2: Tool for pregnant women and their partners about the choice of induction of labor at 41 weeks and expectant management. Tool 3: Tool for pregnant women and their partners about alternative options when choosing expectant management. [Colour figure can be viewed at wileyonlinelibrary.com]

### 3.3 Content of SDM

Figure 2 illustrates the topics and items that care providers reported as being part of most (defined as 75% or more) of their SDM conversations, based on the BRAINS framework. Figure S6 shows the same items, stratified by professional group. The majority of participants adhered to the recommendations and included most items in their conversations. However, some items had lower adherence rates: Only 37% provided percentages of outcomes for each decision option and 33% discussed differences in outcomes between nulliparous and multiparous women. Alternatives such as artificial rupture of membranes before labor onset and EM beyond 42 weeks were discussed by 58% and 37% respectively, with primary care midwives more likely to provide information about alternatives. Asking women whether a conversation about management of late‐term gestations is desired was performed by 45% of participants, with a higher percentage (59%) among primary care midwives.

**FIGURE 2:**
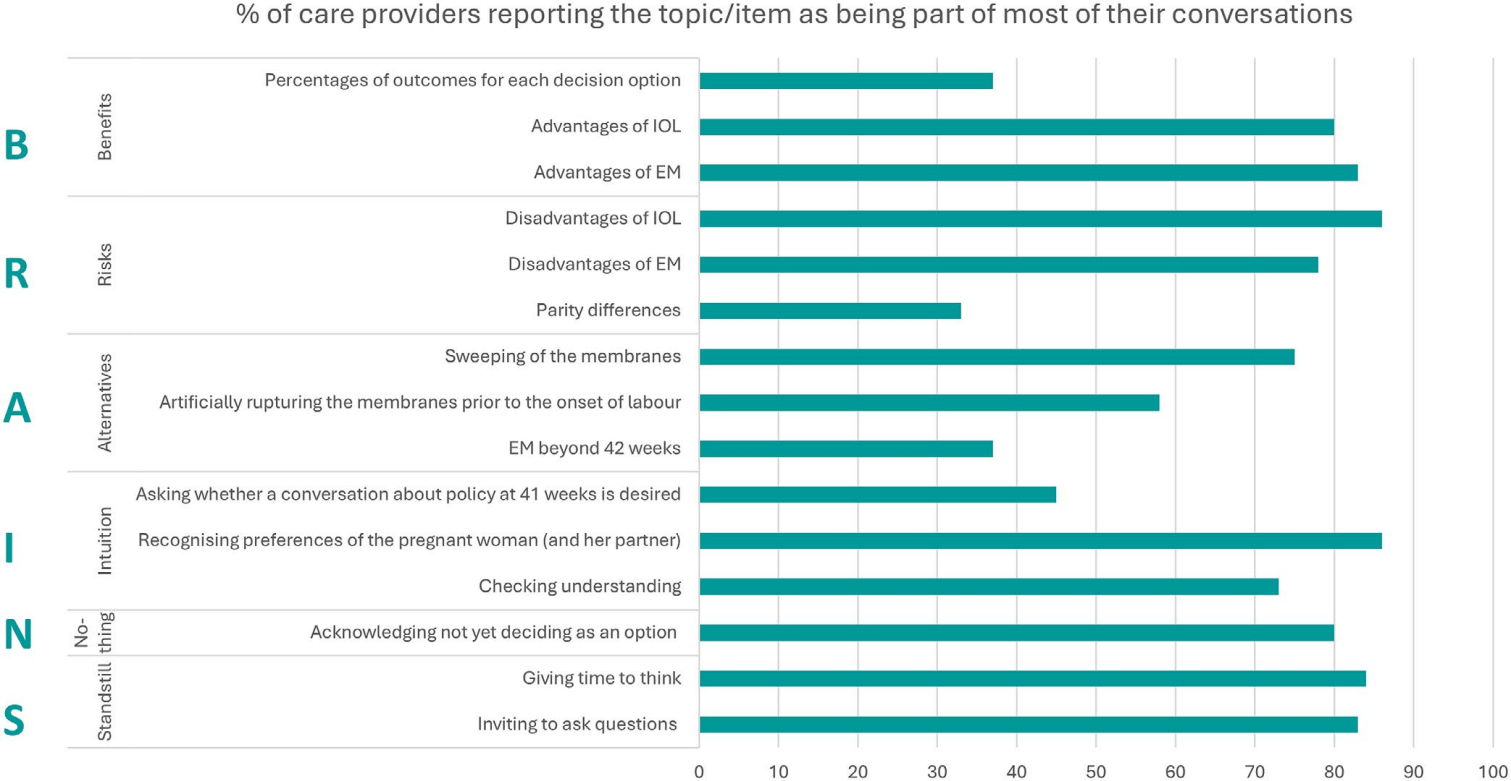
Topics and items that care providers reported as being part of most* of their shared decision‐making on policy at 41 weeks of gestation (*n* = 575). Legend: These topics and items are part of the BRAINS‐method in Tool 1, which prescribes topics and items that should be included in the shared decision‐making conversations. *most = 75% or more. [Colour figure can be viewed at wileyonlinelibrary.com]

### 3.4 Perspectives of SDM

Most care providers reported not emphasizing the benefits of either IOL or EM. However, in multiparous women, the advantages of EM were more often highlighted (41%) (Figure 3). Obstetricians more frequently emphasized the advantages of IOL, while primary care midwives tended to emphasize the benefits of EM (Figure S7). All professional groups scored highly on the importance of the woman's preference being the most important topic in conversations (89%) and decision‐making (97%). Nonetheless, 65% of participants reported that pregnant women often follow their advice because they trust the expertise of the care providers. Only 29% of participants indicated that pregnant women rarely or never had difficulty translating the information into a decision that aligns with their personal values; the rest reported this occurred sometimes (57%) or often/always (14%). Not completing the full SDM session in cases of language barriers or functional illiteracy was reported as sometimes by 43% and often/always by 22%. Primary care midwives more frequently reported difficulties in conversing about the risk of perinatal mortality, followed by hospital‐based midwives and obstetricians, who rarely reported this.

**FIGURE 3:**
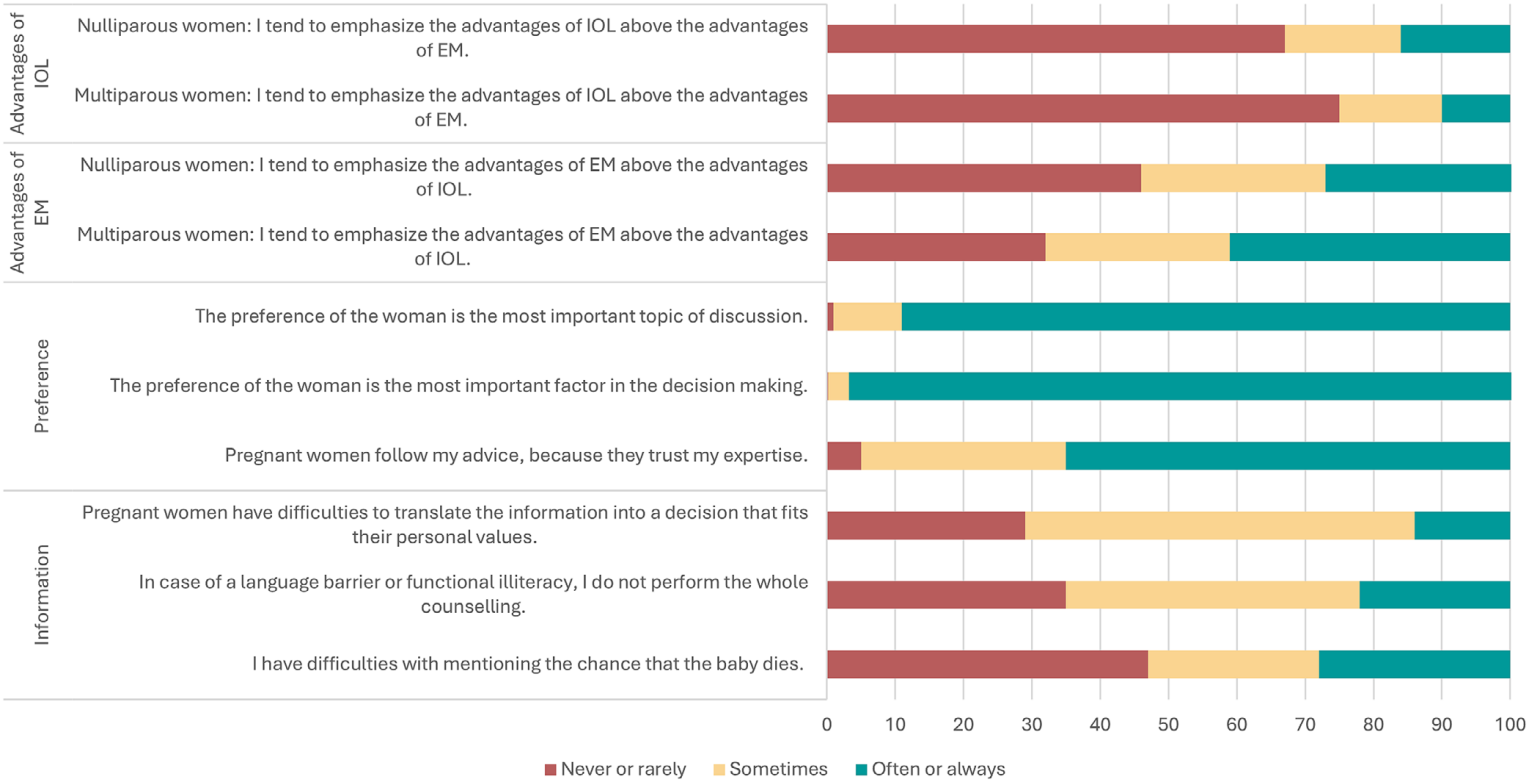
Care providers' perspectives of shared decision‐making, rated with a 5‐point Likert scale (*n* = 518). Legend: 5‐point Likert Scales were used. Likert Scale options: Never—rarely—sometimes—often—always. [Colour figure can be viewed at wileyonlinelibrary.com]

### 3.5 Association of BBS With Content and Perspectives of SDM

Tables 2 and 3 show the associations between care providers' birth beliefs with self‐reported topics and items in SDM conversations (Table 2) and their perspectives on SDM (Table 3). As topics and items were measured dichotomously, the associations are presented using odds ratios (OR), while perspectives are presented as regression coefficients (β) as these were measured using 5‐point Likert scores.

**TABLE 2:** Association between Birth Beliefs Scale and self‐reported topics and items in SDM conversations (*n* = 483).

Topics/items discussed in most* SDM conversations			Medical view (Birth Beliefs Scale)			Natural view (Birth Beliefs Scale)		
			Mean [SD]	**OR** (95% CI)	a**OR** ** (95% CI)	Mean (SD)	OR (95% CI)	a**OR** ** (95% CI)
**Benefits**	Percentages of outcomes for each decision option	Yes	2.86 [0.61]	**1.39** **(1.02–1.89)**	0.88 (0.58–1.33)	3.76 (0.66)	**0.64** (0.48–0.85)	0.95 (0.64–1.41)
		No	2.74 [0.59]	—	—	3.94 (0.61)	—	—
	Advantages of IOL	Yes	2.85 [0.59]	**4.44** **(2.69–7.35)**	**2.62** **(1.39–4.94)**	3.82 (0.61)	**0.31** **(0.18–0.54**)	0.73 (0.39–1.36)
		No	2.32 [0.55]	—	—	4.21 (0.68)	—	—
	Advantages of EM	Yes	2.77 [0.60]	0.60 (0.35–1.04)	0.90 (0.42–1.89)	3.90 (0.62)	**2.17** **(1.36–3.48)**	**1.87** **(1.02–3.43)**
		No	2.95 [0.68]	—	—	3.56 (0.74)	—	—
**Risks**	Disadvantages of IOL	Yes	2.81 [0.59]	**2.23** **(1.19–4.18)**	2.22 (0.98–5.05)	3.88 (0.61)	1.47 (0.83–2.63)	**2.13** **(1.05–4.33)**
		No	2.51 [0.81]	—	—	3.71 (0.94)	—	—
	Disadvantages of EM	Yes	2.85 [0.59]	**2.90** **(1.87–4.50)**	**1.99** **(1.13–3.51)**	3.82 (0.62)	**0.38** **(0.24–0.61)**	0.64 (0.35–1.18)
		No	2.46 [0.60]	—	—	4.16 (0.67)	—	—
	Parity differences	Yes	2.87 [0.59]	**1.40** **(1.02–1.91)**	1.27 (0.83–1.95)	3.78 (0.66)	**0.70** **(0.52–0.94)**	0.80 (0.54–1.20)
		No	2.75 [0.61]	—	—	3.92 (0.62)	—	—
**Alternatives**	Sweeping of the membranes	Yes	2.71 [0.57]	**0.22** (0.13–0.35)	0.72 (0.37–1.40)	3.98 (0.56)	**6.01** **(3.78–9.58)**	**2.78** **(1.63–4.74)**
		No	3.18 [0.64]	—	—	3.32 (0.68)	—	—
	Artificially rupturing the membranes prior to the onset of labor	Yes	2.66 [0.57]	**0.33** (0.23–0.47)	0.89 (0.56–1.43)	4.01 (0.56)	**2.79** **(2.00–3.87)**	1.07 (0.70–1.66)
		No	3.02 [0.60]	—	—	3.62 (0.68)	—	—
	EM beyond 42 weeks	Yes	2.62 [0.58]	**0.46** (0.33–0.63)	0.70 (0.46–1.07)	4.08 (0.54)	**2.70** **(1.93–3.77)**	**2.02** **(1.30–3.15)**
		No	2.90 [0.60]	—	—	3.72 (0.65)	—	—
**Intuition**	Asking whether counseling is desired	Yes	2.63 [0.61]	**0.38** **(0.28–0.53)**	0.69 (0.45–1.06)	4.05 (0.62)	**2.72** **(1.97–3.76)**	**1.66** **(1.10–2.51)**
		No	2.95 [0.56]	—	—	3.69 (0.60)	—	—
	Recognizing preferences of the pregnant woman (and her partner)	Yes	2.80 [0.59]	1.52 (0.75–3.07)	2.04 (0.82–5.10)	3.88 (0.61)	**2.04** **(1.09–3.80)**	**2.87** **(1.34–6.16)**
		No	2.64 [0.83]	—	—	3.56 (1.00)	—	—
	Checking understanding	Yes	2.81 [0.60]	1.34 (0.92–1.94)	0.87 (0.53–1.44)	3.85 (0.61)	0.84 (0.58–1.20)	1.29 (0.81–2.08)
		No	2.71 [0.64]	—	—	3.92 (0.72)	—	—
**Nothing**	Acknowledging not yet deciding as an option	Yes	2.75 [0.59]	**0.35** **(0.20–0.59)**	0.61 (0.30–1.25)	3.93 (0.59)	**3.41** **(2.16–5.37)**	**2.93** **(1.62–5.29)**
		No	3.10 [0.62]	—	—	3.41 (0.76)	—	—
**Standstill**	Giving time to think	Yes	2.78 [0.60]	0.71 (0.40–1.26)	0.82 (0.37–1.80)	3.89 (0.62)	**2.06** **(1.25–3.40)**	**2.41** **(1.28–4.53)**
		No	2.91 [0.70]	—	—	3.57 (0.77)	—	—
	Inviting to ask questions	Yes	2.79 [0.61]	1.08 (0.63–1.86)	0.66 (0.32–1.38)	3.87 (0.62)	1.03 (0.61–1.72)	1.67 (0.90–3.12)
		No	2.76 [0.62]	—	—	3.86 (0.83)	—	—

* Most = 75% or more.

** Adjusted for profession, frequency of SDM conversations, years of experience, gender, and age.

**TABLE 3:** Association between Birth Beliefs Scale and care providers' perspectives on SDM regarding policy at 41 weeks of gestation (*n* = 483).

Perspectives on SDM	Mean [SD]	Medical view (Birth Beliefs Scale)		Natural view (Birth Beliefs Scale)	
		β (95% CI)	aβ* (95% CI)	β (95% CI)	aβ* (95% CI)
Nulliparous: Emphasize the advantages of IOL above the advantages of EM	2.14 [1.15]	**1.01** **(0.87–1.16)**	**0.42** **(0.24–0.59)**	**−0.87** **(−1.01 to −0.73)**	**−0.20** **(−0.37 to −0.04)**
Multiparous: Emphasize the advantages of IOL above the advantages of EM	1.93 [1.03]	**0.91** **(0.78–1.04)**	**0.38** **(0.22–0.53)**	**−0.87** **(−0.99 to −0.75)**	**−0.35** **(−0.50 to −0.20)**
Nulliparous: Emphasize the advantages of EM above the advantages of IOL	2.66 [1.15]	**−0.70** **(−0.86 to −0.54)**	**−0.27** **(−0.47 to −0.07)**	**0.71** **(0.56–0.86)**	**0.30** **(0.11–0.49)**
Multiparous: Emphasize the advantages of EM above the advantages of IOL	3.01 [1.19]	**−0.70** **(−0.86 to −0.53)**	**−0.27** **(−0.49 to −0.06)**	**0.80** **(0.64–0.95)**	**0.48** **(0.28–0.68)**
The preference of the woman is the most important topic of discussion	4.29 [0.69]	**−0.16** **(−0.26 to −0.06)**	−0.04 (−0.18 to 0.09)	**0.25** **(0.16–0.35)**	**0.23** **(0.11–0.36)**
The preference of the woman is the most important topic of decision‐ making	4.52 [0.57]	**−0.15** **(−0.23 to −0.06)**	**−0.11** **(−0.22 to −0.00** **)	**0.25** **(0.17–0.33)**	**0.31** **(0.21–0.42)**
Pregnant women follow my advice because they trust my expertise	3.59 [0.68]	**0.18** **(0.08–0.28)**	0.12 (−0.02 to 0.25)	**−0.10** **(−0.19 to −0.00** ***)	0.04 (−0.09 to 0.17)
Pregnant women have difficulties to translate the information into a decision that fits their personal values	2.84 [0.68]	−0.02 (−0.12 to 0.08)	0.03 (−0.10 to 0.17)	0.05 (−0.05 to 0.15)	0.02 (−0.11 to 0.15)
In case of a language barrier or functional illiteracy, I do not perform the whole counseling	2.78 [0.89]	−0.05 (−0.18 to 0.08)	0.01 (−0.16 to 0.19)	0.09 (−0.04 to 0.21)	0.06 (−0.11 to 0.23)
I have difficulties with mentioning the chance that the baby dies	2.64 [1.26]	**−0.48** **(−0.67 to −0.30)**	0.06 (−0.17 to 0.29)	**0.49** **(0.31 to 0.66)**	−0.04 (−0.26 to 0.19)

* Adjusted for profession, frequency of SDM conversations, years of experience, gender, and age.

** −0.00 = −0.0001601.

*** −0.00 = −0.0003972.

Care providers with higher medical BBS scores were generally more intervention‐oriented. They more often discussed the advantages of IOL (aOR 2.62; 95% CI 1.39–4.94) and the disadvantages of EM (aOR 1.99; 95% CI 1.13–3.51) (Table 2). They were also more likely to emphasize the advantages of IOL over EM, particularly for nulliparous women (aβ 0.42; 95% CI 0.24–0.59) (Table 3). In contrast, providers with higher natural BBS scores showed the opposite pattern. They more frequently discussed the advantages of EM (aOR 1.87; 95% CI 1.02–3.43) and disadvantages of IOL (aOR 2.13; 95% CI 1.05–4.33) (Table 2). When asked about emphasizing EM over IOL, natural BBS scores were positively associated (aβ 0.30; 95% CI 0.11–0.49), and medical scores negatively (aβ −0.27; 95% CI −0.47 to −0.07) (Table 3).

Care providers with higher natural BBS scores also more often mentioned the alternative options such as sweeping of membranes (aOR 2.78; 95% CI 1.63–4.74) and EM beyond 42 weeks (aOR 2.02; 95% CI 1.30–3.15) (Table 2).

Although medical BBS scores were not associated with items within the themes Intuition, Nothing, and Standstill, higher natural BBS scores were associated with higher odds for asking whether counseling is desired (aOR 1.66; 95% CI 1.10–2.51), recognizing preferences (aOR 2.87; 95% CI 1.34–6.16), acknowledging not yet deciding as an option (aOR 2.93; 95% CI 1.62–5.29), and giving time to think (aOR = 0.61, 95% CI [0.30, 1.25]) (Table 2). This was also reflected in their perspectives (Table 3): Higher medical BBS scores were negatively associated with prioritizing the woman's preference in decision‐making (aβ −0.11; 95% CI −0.22 to –0.00), whereas natural BBS scores were positively associated with doing so (aβ 0.31; 95% CI 0.21–0.42).

## 4 Discussion

### 4.1 Main Findings

Awareness of the tools provided with the guideline on late‐term gestation management was generally lower than awareness of the guideline itself, with many care providers either unaware of the tools or not using them. Primary care midwives, compared to obstetricians and hospital‐based midwives, more frequently used the tool incorporating the BRAINS steps and non‐guideline tools. Obstetricians more often used the choice card related to IOL and EM. Overall, care providers were positive about the tools. While many included various BRAINS steps in SDM conversations, outcome percentages, parity‐based differences, and alternatives were often omitted. Most care providers reported considering the woman's preferences but noted challenges with language barriers and clients struggling to make informed decisions. Regardless of profession, care providers' beliefs influence both the content and perspective of SDM conversations. Providers with a stronger medical view tend to emphasize the advantages of IOL, whereas those with a stronger natural view more often tend to highlight the benefits of EM. Greater attention to women's preferences, alternative options, and allowing time to consider decisions was consistently associated with a more natural view.

### 4.2 Interpretation

Most items recommended for inclusion in SDM conversations on late‐term management were incorporated by the majority of care providers, but there were notable deviations. Less than half of the providers reported mentioning percentages of outcomes for each decision option, differences between nulliparous and multiparous women, or EM beyond 42 weeks, although these are being recommended by the national guideline [11]. Literature highlights that quantitative information about chances is crucial for understanding [18]. In our study, 71% of providers noted that pregnant women had difficulties translating the information into decisions that aligned with their values, consistent with evidence of inadequate health literacy among many pregnant women [19, 20]. Omitting percentages can result in subjective messaging and variation between care providers, as qualitative terms such as ‘more’ and ‘less’ involve value judgments from both parties [18, 21]. This applies to women with both adequate and inadequate health literacy [20]. Presenting numbers as natural frequencies, such as 4 out of 100 instead of 4%, with supporting visual aids, especially icon arrays, can improve understanding [18, 21].

The choice cards in the guideline do use natural frequencies but lack accompanying visual aids. Although a video featuring icon arrays was developed by the guideline working group, it was released after the survey and could not be assessed in this study. In our findings, 19% of the care providers were unaware of the choice card outlining the pros and cons of IOL and EM, and 28% of those who were aware did not use it. This raises concerns about whether all pregnant women and their partners are receiving sufficient information for a thorough SDM process.

Furthermore, the four SDM steps described by Stiggelbout et al. [2] (Textbox 1) are all encompassed in the BRAINS steps recommended for conversations [2]. Using this tool can ensure that all essential SDM elements are included. This may also improve adherence to discussing the differences between nulliparous and multiparous women. This is crucial, as the study of Alkmark et al. [22] lacked the power to detect a difference in multiparous women regarding severe adverse perinatal outcomes and did not show a significant benefit of IOL at 41 weeks for multiparous women [22]. Additionally, discussing choice alternatives is necessary, but only 37% of providers reported discussing EM beyond 42 weeks, and the choice card with alternatives was underused. However, 29% of participants reported using non‐guideline tools, which may include the video released after the survey or other non‐working group tools. Further research is needed to explore care providers' and pregnant women's preferences for the format and content of choice cards for late‐term management.

Most care providers reported prioritizing the woman's perspective as the most important factor in conversations and decision‐making. At the same time, 65% indicated that pregnant women often or always follow the care provider's advice because they trust their expertise, highlighting the significant influence care providers have on decision‐making.

Obstetricians more often emphasized the benefits of IOL, whereas primary care midwives more frequently highlighted the advantages of EM and more often reported discussing alternative options. Regardless of professional background, care providers' birth beliefs also shaped how they framed these options. These findings suggest that beyond professional background, care providers' underlying views on pregnancy and childbirth shape how they frame options and conduct SDM conversations. While the national guideline recommends presenting IOL and EM as equally valid options [11], such variation in emphasis can impact how women perceive their choices. When alternatives are not actively addressed, women may consent to IOL without fully understanding their options. This can undermine informed SDM and shift the conversation towards a provider‐led rather than preference‐based approach. Our findings further suggest that a stronger medical view may relate to less attention to women's preferences and less time for decision‐making. Care providers should therefore be aware of how their personal beliefs influence the SDM process and ensure that decision‐making is guided primarily by women's preferences [23, 24, 25, 26, 27]. A well‐conducted SDM conversation can contribute to a more positive birth experience [4, 23, 28]. Women consistently express a desire to be actively involved in decisions about IOL, supporting their sense of autonomy [2, 4].

### 4.3 Strengths and Limitations

A major strength in this study was the substantial sample size with diverse representation across various professional groups. The extensive nature of the questionnaire allowed for valuable insights into the content of the conversations and the views of care providers.

This study has some limitations. A potential recall bias may have influenced the results, as participants were asked to indicate which items were present in their SDM conversations. Besides, care providers may have been inclined to give socially desirable answers. These issues may have led to an overestimation of socially desirable responses, and therefore the actual adherence to the recommendations on SDM may be even lower. Selection bias might also be present, given that participants were probably more engaged with the guideline's topic than the average care provider. However, it is uncertain whether this bias predominantly involved more critical providers or those with a more favorable view of the guideline. Because we included participants' characteristics at the end of the questionnaire, incomplete surveys mainly limited their inclusion in the analyses of associations between characteristics and outcomes. Last, we have examined SDM from the perspective of care providers and therefore are unable to draw conclusions regarding the women's perspective.

## 5 Conclusions

Although care providers were generally positive about the tools provided with the Dutch guideline on late‐term management, not all of them make use of these tools. Additionally, key details about late‐term management, which are essential for SDM, are often left out of conversations. Care providers also report encountering barriers to enabling women to make informed decisions. To optimize SDM in late‐term pregnancy management, it is essential that care providers include all the essential items in the conversation and consistently include quantitative information, such as outcome percentages, parity differences, and alternatives. These elements are recommended in the national guideline, which provides a tool to facilitate this, although it is currently underutilized. Additionally, using visual aids is key to improving understanding for women with both low and high literacy levels. Furthermore, care providers should be aware of their own beliefs during conversations, as these may vary and significantly influence women's choices. In SDM, the woman's preferences should be central, as this supports their autonomy and contributes to a positive birth experience.

## Author Contributions

**Kika Grosheide:** formal analysis, data curation, writing – original draft. **Hannah de Klerk:** conceptualization, writing – review and editing. **Corine J. Verhoeven:** conceptualization, writing – review and editing, supervision. **Caroline J. Bax:** conceptualization, writing – review and editing. **Judit K. J. Keulen:** conceptualization, writing – review and editing. **Lilian L. Peters:** conceptualization, writing – review and editing. **Fatima Hammiche:** conceptualization, writing – review and editing. **Anna E. Seijmonsbergen‐Schermers:** conceptualization, investigation, funding acquisition, writing – original draft, methodology, visualization, formal analysis, project administration.

## Funding

This study was funded by the Koninklijke Nederlandse Organisatie van Verloskundigen, the Dutch Professional Organization of Midwives (project no. 2012862). In addition, there was internal funding from Inholland University of Applied Sciences, Midwifery Academy Amsterdam Groningen (AVAG). The corresponding author had full access to all the data in the study and had final responsibility for the decision to submit for publication. The funders had no role in study design, data collection and analysis, decision to publish, or preparation of the manuscript.

## Conflicts of Interest

Hannah de Klerk (midwife) and Caroline Bax (obstetrician) were members of the working group that developed the Dutch national guideline on management of pregnancies at 41 weeks' gestation onwards. This involvement helped ensure correct interpretation of the guideline and enhance the practical value of the study's findings.

## Supporting information


**File S1:** Questionnaire LATE‐study.

**File S2:** Tool 1.

**File S3:** Tool 2.

**File S4:** Tool 3.

**Figure S5:** Care providers' opinion of tools* provided with the guideline, rated with a 5‐point Likert Scale, stratified by profession (*n* = 572) (“Tool is useful/helpful”).
Legend:
5‐point Likert Scales were used.
Likert Scale options: strongly disagree (1) – disagree (2) – neutral (3) – agree (4) – strongly agree (5).
* Tools in the guideline.
Tool 1: Tool for care providers including BRAINS‐elements.
Tool 2: Tool for pregnant women and their partners about the choice of induction of labor at 41 weeks and expectant management.
Tool 3: Tool for pregnant women and their partners about alternative options when choosing expectant management.

**Figure S6:** Topics and items that care providers reported as being part of most* of their shared decision‐making on policy at 41 weeks of gestation, stratified by profession (*n* = 575).
Legend:
These topics and items are part of the BRAINS‐method in Tool 1, which prescribes topics and items that should be included in the shared decision‐making conversations.
*most = 75% or more

**Figure S7:** Care providers' perspectives of shared decision‐making (mean score on a 5‐point Likert scale), stratified by profession (*n* = 518).
Legend:
5‐point Likert Scales were used.
Likert Scale options: never (1) – rarely (2) – sometimes (3) – often (4) – always (5).

## Data Availability

The data that support the findings of this study are openly available in DANS data Station Life Sciences at https://doi.org/10.17026/LS/KZMWQT, reference number doi:10.17026/LS/KZMWQT.
