# Supplementary material for: Shared Decision‐Making on Late‐Term Gestation Management (LATE‐Study): A Cross‐Sectional Survey Among Maternity Care Providers
Source: Birth. 2026 Aug 2;53(4):819–31. doi: 10.1111/birt.70099 (PMC13728515; doi:10.1111/birt.70099)
Supplement: Supplementary file 1 [file BIRT-53-819-s001.pdf]

Counseling volgens BRAINS-methode

Bij de counseling van zwangeren over het beleid na 41 weken zwangerschap is het belangrijk dat onderstaande punten objectief en volledig met de zwangere vrouw besproken worden (volgens BRAINS-methode).

- Er zijn drie beleidsopties onderzocht voor laag risico zwangeren: het afwachten van een spontane bevalling tot 41 weken zwangerschap, tot 42 weken zwangerschap of tot langer dan 42 weken zwangerschap. De zwangere vrouw kiest zelf welke optie ze prefereert. Dit kan ook een tussenoplossing zijn zoals afwachten tot 41+3 weken.

B: Beweegredenen

Wat zijn de voordelen van inleiden bij 41 weken?

- Inleiden bij een zwangerschapsduur van 41 weken verkleint het risico op perinatale sterfte. Het absolute risico op sterfte is 3 per 1000 (0,3%). Bij inleiden is er minder kans dat de baby opgenomen moet worden in het ziekenhuis na de bevalling (afwachtend beleid: 41 per 1000 (4,1%) versus inleiden: 28 per 1000 (2,8%)).
- Dit effect is mogelijk groter bij vrouwen die zwanger zijn van hun eerste kind in vergelijking met vrouwen die eerder zijn bevallen (Alkmark, 2020). Die kinderen moeten vaker opgenomen worden.

Wat zijn de voordelen van afwachten tot 42 weken zwangerschap?

- Bij afwachtend beleid worden er waarschijnlijk minder medische ingrepen uitgevoerd en vrije keuze voor plaats bevalling (indien van toepassing).
- Bij afwachtend beleid is de kans 74 tot 77 per 100 (74 tot 77%) dat de bevalling spontaan op gang komt tussen 41 en 42 weken zwangerschap (Keulen, 2019). Gemiddeld bevallen vrouwen met een zwangerschapsduur van meer dan 41 weken bij 41+2 weken zwangerschap.

- Er wordt geen significant verschil gezien in (het aantal):
  - Keizersnedes.
  - Vaginale kunstverlossing.
  - Neonatale infecties.
  - Postpartum bloedverlies.
  - Schouderdystocie.
  - Rupturen.
  - Apgar score < 7 na 5 minuten.
- Er werd wel een significante toename van gebruik van pijnstilling gezien bij de groep die werd ingeleid, echter was dit geen klinisch relevant verschil.

R: Risico's

Welke risico's/nadelen zijn er bekend van inleiden?

- De bevalling komt niet spontaan op gang, maar wordt kunstmatig met medicijnen of mechanische middelen op gang gebracht. Hierdoor duurt een inleiding soms langer. Zowel bij inleiden als bij afwachten (tussen 41 en 42 weken) lukt in circa 11 per 100 (11%) een vaginale baring niet. Voor nullipara is dat 18 per 100 (18%) en voor multipara is dat 2 per 100 (2%) (Alkmark, 2020).
- De bevalling vindt vrijwel altijd in het ziekenhuis plaats. Verschillen met een bevalling in de 1e lijn zijn medische ingrepen als CTG-bewaking, soms een infuus en mogelijk meer inwendige onderzoeken.
- Er is geen onderzoek gedaan naar de effecten van inleiding bij 41 weken op de lange termijn, zoals de effecten op de gezondheid van kinderen, maternale psyche en borstvoeding.

Welke risico's/nadelen zijn er bekend van afwachten

- Bij afwachtend beleid wordt er individueel afgestemd hoe vaak er een controle plaats vindt tot 42 weken zwangerschap. Tot op heden is het niet duidelijk of extra controles met CTG en/of echoscopisch onderzoek slechte uitkomsten kunnen voorspellen.

A: Alternatieven

Er zijn meerdere opties voor een alternatief beleid en het beleid wordt individueel afgestemd:

- Strippen: hierdoor neemt de kans op een spontane start van de bevalling met 18% toe (NNT 6). Er kan overwogen worden dit voor 41 weken al aan te bieden na bespreken van de procedure en voor- en nadelen.
- AROM in de eerste lijn (indien van toepassing en regionaal beleid): hierdoor is er meer kans op een eerstelijns bevalling met minder medische ingrepen.
- Afwachten tot na 42 weken:
  - Inleiden bij een zwangerschapsduur van 42 weken verkleint het risico op perinatale sterfte in vergelijking met afwachten na 42 weken (afwachtend beleid: 3 per 1000 (0,3%) versus inleiden: 0,3 per 1000 (0,03%)), opname van de baby in het ziekenhuis en risico op een keizersnede. Op basis hiervan wordt zwangeren geadviseerd niet langer af te wachten.
  - Er is geen onderzoek gedaan naar de effecten op lange termijn, zoals de effecten op de gezondheid van de pasgeborene, maternale psyche en borstvoeding.
  - Bij afwachtend beleid wordt er individueel afgestemd hoe vaak er een controle plaats vindt na 42 weken zwangerschap. Tot op heden is het niet duidelijk of extra controle met CTG en/of echoscopisch onderzoek slechte uitkomsten kunnen voorspellen.

I: intuïtie - voorkeuren cliënt

- Begrijp je de informatie?
- Heb je nog meer nodig om een besluit te nemen? (second opinion, extra informatie)
- Wat zegt je intuïtie? Wat denk/voel je nu ik je dit heb verteld?

- Wat is voor jou van belang?
- Wat wil jij?

N: Niks doen - Wat gebeurt er als ik wacht?

- Je hoeft nu nog geen keuze te maken.
- Er is een reële kans dat de bevalling spontaan begint tussen nu en het volgende consult.

S: Stilstaan - volgende consult erop terugkomen

- De zorgverlener:
    - Geeft de zwangere vrouw tijd om de informatie te bespreken met haar partner en/of naasten;
    - Moedigt de zwangere vrouw aan zich goed te informeren met behulp van verschillende bronnen;
    - Nodigt de zwangere vrouw uit om vragen te stellen;
    - Moedigt de zwangere vrouw aan om over verschillende opties na te denken;
    - Ondersteunt de zwangere vrouw in welke beslissing ze ook neemt.
