# Supplementary material for: Shared Decision‐Making on Late‐Term Gestation Management (LATE‐Study): A Cross‐Sectional Survey Among Maternity Care Providers
Source: Birth. 2026 Aug 2;53(4):819–31. doi: 10.1111/birt.70099 (PMC13728515; doi:10.1111/birt.70099)
Supplement: Supplementary file 2 [file BIRT-53-819-s004.pdf]

KEUZEKAART Keuzemogelijkheden vanaf 41 weken zwangerschap

Inleiden of afwachten – deel 1

De normale periode om te bevallen is tussen 37 en 42 weken zwangerschap. Deze keuzekaart kan jou en je zorgverlener helpen je mogelijkheden te bespreken vanaf 41 weken zwangerschap. We gaan er hier vanuit dat er geen medische redenen zijn om je bevalling in te leiden.

Keuzes →	Afwachten tot 42 weken zwangerschap	Inleiden van de bevalling vanaf 41 weken zwangerschap in het ziekenhuis
Wat zijn de risico's voor mijn kind?	De meeste bevallingen verlopen zonder problemen.<br>• 41 van de 1000 baby's (4,1%) worden opgenomen in het ziekenhuis.<br>• 3 van de 1000 baby's (0,3%) overlijden. We weten niet precies waarom.<br>• Deze risico's lijken wat groter bij je eerste kind en kleiner als je al eerder bevallen bent.	De meeste bevallingen verlopen zonder problemen.<br>• 28 van de 1000 baby's (2,8%) worden opgenomen in het ziekenhuis.<br>• 1 van de 1000 baby's (0,1%) overlijdt.<br>• De kans op infectie of een slechte start bij de baby is even groot.<br>• We weten niet of inleiden later invloed heeft op het kind.
Wat zijn de risico's voor mijzelf?	• De kans is kleiner dat je pijnstilling krijgt (46 van de 100).<br>• De kans is waarschijnlijk iets kleiner dat de bevalling langer duurt.<br>• Vergeleken met inleiden, is de kans even groot dat deze problemen ontstaan:<br>- Je bevalt met een vacuümpomp of keizersnede.<br>- Je scheurt in.<br>- Je verliest veel bloed bij de bevalling.	• De kans is groter dat je pijnstilling krijgt (50 van de 100).<br>• De kans is waarschijnlijk iets groter dat de bevalling langer duurt.<br>• Het is niet bekend of je door zelf te kiezen voor inleiden vanaf 41 weken, de bevalling als positiever of negatiever ervaart.<br>• Vergeleken met afwachten, is de kans even groot dat deze problemen ontstaan:<br>- Je bevalt met een vacuümpomp of keizersnede.<br>- Je scheurt in.<br>- Je verliest veel bloed bij de bevalling.
Wat gebeurt er?	• Bij ongeveer 75 van de 100 (75%) vrouwen begint na 41 weken zwangerschap de bevalling vanzelf vóór 42 weken. Deze begint met weeën of doordat de vliezen breken.<br>• Ben je onder controle bij een verloskundige? Dan zal deze je bij je bevalling begeleiden. Je kunt kiezen of je thuis of in het ziekenhuis wilt bevallen.<br>• De verloskundige checkt regelmatig de hartslag van de baby.<br>• Tijdens je bevalling kun je bewegen. Bijvoorbeeld rondlopen of in bad gaan.<br>• De tijd dat je weeën hebt is vaak korter. Vooral als je voor de eerste keer bevalt.	• Voordat de inleiding kan starten moet je al een beetje ontsluiting hebben. Heb je dit nog niet? Dan krijg je een ballonnetje in de baarmoedermond. Of je krijgt medicijnen om te slikken, of in de vagina. Zo kan de baarmoedermond al een beetje open gaan. Dit duurt soms een paar dagen.<br>• Je verblijft voor de inleiding in het ziekenhuis. Je bevalling zelf start doordat de arts of de verloskundige de vliezen breekt. En door een infuus met medicijnen die je weeën opwekken. Krijg je snel weeën nadat de vliezen zijn gebroken? Dan krijg je geen medicijnen die weeën opwekken.<br>• In het ziekenhuis zul je meerdere verpleegkundigen, verloskundigen en artsen zien.<br>• Je kunt moeilijker bewegen, bijvoorbeeld rondlopen of in bad gaan. Dat komt door het infuus dat je in je arm hebt. En door de CTG-band om je buik. Met deze band checkt de arts de hartslag van de baby. Je krijgt vaker een inwendig onderzoek.<br>• De tijd dat je weeën hebt is vaak langer. Vooral als je voor de eerste keer bevalt.
Wat is de kans dat het lukt?	Bij 75 van de 100 vrouwen (75%) begint de bevalling vanzelf vóór 42 weken zwangerschap.	Het lukt bijna altijd om je bevalling op te wekken. Bij een klein aantal vrouwen lukt dit niet. In overleg kun je afwachten, of het later nog een keer proberen of krijg je soms een keizersnede.
Wat is de invloed op de binding tussen moeder en kind?	Het is niet bekend of afwachten invloed heeft op de binding tussen moeder en kind.	Het is niet bekend of inleiden invloed heeft op de binding tussen moeder en kind.

Deze keuzekaart is gemaakt met ondersteuning van het Kennisinstituut van de Federatie Medisch Specialisten en is te vinden op THUISARTS.NL THUIS IN GEZONDHEID
