# Supplementary material for: Shared Decision‐Making on Late‐Term Gestation Management (LATE‐Study): A Cross‐Sectional Survey Among Maternity Care Providers
Source: Birth. 2026 Aug 2;53(4):819–31. doi: 10.1111/birt.70099 (PMC13728515; doi:10.1111/birt.70099)
Supplement: Supplementary file 3 [file BIRT-53-819-s006.pdf]

KNOV | Patiëntenfederatie Nederland – samen de zorg beter maken | nvog – Nederlandse Vereniging voor Obstetrie & Gynaecologie | 2022

KEUZEKAART Keuzemogelijkheden vanaf 41 weken zwangerschap

Andere mogelijkheden – deel 2

De normale periode om te bevallen is tussen 37 en 42 weken zwangerschap. Deze keuzekaart kan jou en je zorgverlener helpen je mogelijkheden te bespreken vanaf 41 weken zwangerschap. We gaan er hiervan uit dat er geen medische redenen zijn om je bevalling in te leiden.

Keuzes ➔	Strippen	Breken van de vliezen door de verloskundige	Afwachten na 42 weken zwangerschap
**Wat zijn de risico's voor mijn kind?**	De kans dat er iets misgaat voor de baby is heel klein.	De kans dat er iets misgaat voor de baby is heel klein.	De kans is groter dat:<br>• Je baby moet worden opgenomen in het ziekenhuis.<br>• Je baby in de buik overlijdt.<br>We weten niet precies hoe vaak dit gebeurt.
**Wat zijn de risico's voor mijzelf?**	De kans dat er iets misgaat bij jezelf is heel klein. Sommige vrouwen ervaren het strippen als onprettig of pijnlijk. Of je hebt erna last van onprettige harde buiken. Na het strippen kun je wat bloed verliezen uit je vagina.	De kans dat er iets misgaat bij jezelf is heel klein.	• De kans dat er iets misgaat bij jezelf is heel klein.<br>• De kans is groter dat je een keizersnede krijgt.
**Wat gebeurt er?**	Je verloskundige of arts voelt met de vingers in je vagina of je al wat ontsluiting hebt. Dus of de baarmoedermond al een beetje open staat.<br>Is dit zo? Dan maakt zij de vliezen aan de binnenkant van de baarmoedermond los. Dit noemen we strippen. Ze breekt de vliezen niet. Door het strippen komen hormonen vrij die kunnen helpen de bevalling op gang te brengen. Het strippen kan worden herhaald.	De bevalling start doordat je verloskundige de vliezen breekt. Je wacht dan een aantal uur af of de weeën daarna vanzelf beginnen. Beginnen de weeën vanzelf? Dan beval je met je verloskundige, waar jij wil: thuis of in het ziekenhuis. Beginnen ze niet vanzelf? Dan ga je naar het ziekenhuis om alsnog een infuus met medicijnen te krijgen die weeën opwekken.	Ben je meer dan 42 weken zwanger? Dan is de aanbeveling om in het ziekenhuis te bevallen. Ook is de aanbeveling om vanaf 42 weken zwangerschap regelmatig de baby in de gaten te houden met een hartfilmpje. Ook tijdens de bevalling maakt de arts of verloskundige een hartfilmpje van de baby.
**Wat is de kans dat het lukt?**	Bij ongeveer 18 op de 100 vrouwen (18%) begint de bevalling door het strippen vóór 42 weken zwangerschap.	Lukt het om de vliezen te breken? Dan beginnen bij 90 op de 100 vrouwen (90%) de weeën vanzelf.	We weten niet hoeveel dagen het duurt voordat de bevalling vanzelf begint als je afwacht na 42 weken zwangerschap.
**Wat is de invloed op de binding tussen moeder en kind?**	We weten niet of strippen invloed heeft op de binding tussen moeder en kind.	We weten niet of het breken van de vliezen invloed heeft op de binding tussen moeder en kind.	We weten niet of afwachten na 42 weken zwangerschap invloed heeft op de binding tussen moeder en kind.

Deze keuzekaart is gemaakt met ondersteuning van het Kennisinstituut van de Federatie Medisch Specialisten en is te vinden op THUISARTS.NL THUIS IN GEZONDHEID
